# First Record of *Hepatozoon* spp. in Alpine Wild Rodents: Implications and Perspectives for Transmission Dynamics across the Food Web

**DOI:** 10.3390/microorganisms10040712

**Published:** 2022-03-25

**Authors:** Giulia Ferrari, Matteo Girardi, Francesca Cagnacci, Olivier Devineau, Valentina Tagliapietra

**Affiliations:** 1 Fondazione Edmund Mach, Research and Innovation Centre, Via Edmund Mach 1, 38010 San Michele all’Adige, Italy; matteo.girardi@fmach.it (M.G.); francesca.cagnacci@fmach.it (F.C.); valentina.tagliapietra@fmach.it (V.T.); 2 Faculty of Applied Ecology, Agricultural Science and Biotechnology, Campus Evenstad, Inland Norway University of Applied Sciences, 2480 Koppang, Norway; olivier.devineau@inn.no

**Keywords:** *Hepatozoon* spp., *Chionomys nivalis*, *Apodemus* spp., *Myodes glareolus*, Italian Alps

## Abstract

Among the Apicomplexa parasites, *Hepatozoon* spp. have been mainly studied in domestic animals and peri-urban areas. The epidemiology of *Hepatozoon* spp. is poorly investigated in natural systems and wild hosts because of their scarce veterinary and economic relevance. For most habitats, the occurrence of these parasites is unknown, despite their high ecosystemic role. To fill this gap for alpine small mammals, we applied molecular PCR-based methods and sequencing to determine the *Hepatozoon* spp. in 830 ear samples from 11 small mammal species (i.e., *Apodemus*, *Myodes*, *Chionomys*, *Microtus*, *Crocidura* and *Sorex* genera) live-trapped during a cross-sectional study along an altitudinal gradient in the North-Eastern Italian Alps. We detected *Hepatozoon* spp. with an overall prevalence of 35.9%. Two species ranging from 500 m a.s.l. to 2500 m a.s.l. were the most infected: *My. glareolus*, followed by *Apodemus* spp. Additionally, we detected the parasite for the first time in another alpine species: *C. nivalis* at 2000–2500 m a.s.l. Our findings suggest that several rodent species maintain *Hepatozoon* spp. along the alpine altitudinal gradient of habitats. The transmission pathway of this group of parasites and their role within the alpine mammal community need further investigation, especially in consideration of the rapidly occurring environmental and climatic changes.

## 1. Introduction

Protozoa parasites are a highly diverse group of successful organisms globally distributed via a wide range of hosts [1] and whose role is crucial for ecosystem functioning [2]. Among the blood protozoa parasites, the phylum Apicomplexa has received much attention for its zoonotic relevance, for example, the vector borne diseases due to *Plasmodium* spp. or the *Toxoplasma* spp. transmitted in urban cycles that include domestic species and humans. Other protozoan parasites, such as the *Hepatozoon* genus represented by 340 species, have also been commonly recorded in peri-urban cycles sustained by domestic species (e.g., cats and dogs) [3,4,5,6,7,8], although no zoonotic relevance has been reported so far [9,10]. Further, *Hepatozoon* spp. have been detected in amphibians, reptiles, birds that act as intermediate hosts, and mammals, which can be both paratenic and reservoir hosts [11].

The transmission of *Hepatozoon* spp. can occur through different modalities that most often involve the ingestion of infected vectors or hosts. In mammals, for example, animals can become infected through grooming behavior [12] by eating infected arthropod ectoparasites attached to another individual, such as ticks [13], fleas [14], and mosquitoes [15]. Further, predator–prey food web transmission routes can occur through the ingestion of prey either containing infective meronts or infested by infected vectors [16]. Additionally, vertical transplacental transmission has also been reported [17].

*Hepatozoon* spp. have been found in a diversity of habitats across the globe, such as temperate broad-leaved forests [18,19], conifer forests [20], Mediterranean scrubland [21], subtropical forests and savannah [22], and taiga [23], likely indicating complex transmission cycles that are still largely unknown. However, the occurrence and prevalence of *Hepatozoon* spp. in some crucial ecosystems remain unexplored, for example, across altitudinal gradients where the rapid succession of habitats often corresponds to the high biodiversity of partially sympatric hosts. Under global and climate changes, such mountain habitats are particularly exposed to abiotic and biotic variations that favor upward distributional shifts of host mammal species, with local modifications of the communities [24,25]. This may result in parasite expansion in terms of geographic distribution and diversity of the host species involved [26,27]. The monitoring of the occurrence of a parasite with a very plastic transmission cycle, such as *Hepatozoon* spp., is therefore of crucial importance in these habitats undergoing intense dynamics. This is the case for Alpine ecosystems, where *Hepatozoon* spp. were identified in wild carnivores [18,20] but have never been recorded in alpine small mammals [28]. The occurrence of the parasite in the small mammal community is of particular interest, as it prevalently depends on arthropods acting as vectors, the vertical transmission route being secondary. The main arthropods transmitting *Hepatozoon* spp. to rodents are blood ectoparasites, such as ticks, fleas, mites, and mosquitoes [29], the occurrence of which is shifting upward in the Alps as the temperature and humidity conditions become milder with the climate change [30].

In small mammals, *Hepatozoon* spp. life cycles involve schizogony, which occurs in various organs, such as muscles, lymph nodes, the spleen, and generally in the liver, while gametogony occurs in lymphocytes, monocytes, or occasionally, in granulocytes [29]. Further development requires an arthropod vector in which fertilization and sporogony take place [29]. Several species of the *Hepatozoon* genus were detected in European rodents, e.g., *H. lavieri* [31], *H. erhardovae* [23,32], *H. sylvatici* [33,34], *H. griseisciuri* [33], and sporadically *H. muris* (formerly known as *H. perniciosum*) [33,35]. In rodents, the infection of *Hepatozoon* spp. may be pathogenic [36,37,38,39,40], although generally mild and asymptomatic [41]. Conversely, in carnivores (i.e., secondary hosts), such as canids [42], felids [43], mustelids [44], and in snakes [45], *Hepatozoon* spp. may compromise the immune status, thus predisposing it toward coinfection by other pathogens.

In this work, we conducted a cross-sectional study, i.e., we assessed the parasite prevalence without specifically considering the temporal pattern from 2019 to 2021 in the Italian Alps by intensively live-trapping small mammals from forested habitats up to above the tree line. Our main aim was to evaluate the occurrence of *Hepatozoon* spp. across the altitudinal gradient of the Alpine habitat also in consideration of the anthropic pressure on such habitat. To the best of our knowledge, this is the first study investigating *Hepatozoon* spp. occurrence in small mammals, and more generally in rodents in Italy, with a special focus on the Alpine habitat.

## 2. Materials and Methods

### 2.1. Study Area and Animal Sampling

**Figure 1:**
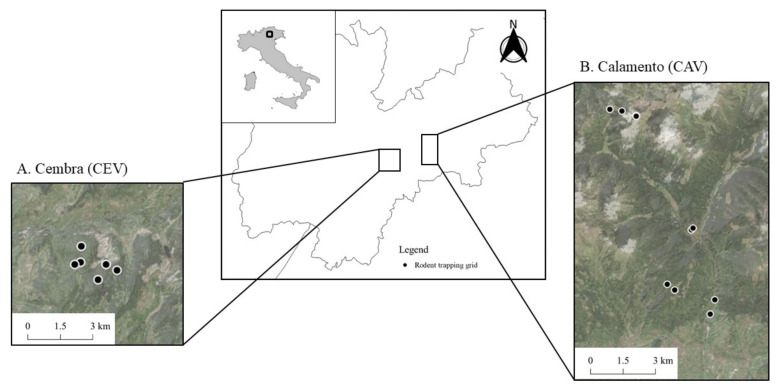
Map of the study sites located in the Province of Trento (Italy). (**A**) Cembra Valley (CEV), where square grids (8 × 8 traps) were placed at 1000 m a.s.l. (**B**) Calamento Valley (CAV), where cross-shaped grids (16 traps) were placed from 500 to 2500 m a.s.l. Black circles show trapping grid locations.

The study was carried out in two sites located in the Province of Trento (Italy): Cembra Valley (CEV; 46.13020 N–11.17843 E, altitude 1000 m a.s.l.) and Calamento Valley (CAV; 46.12092 N–11.48842 E, altitude from 500 to 2500 m a.s.l.) (Figure 1). CEV comprises peri-urban areas with an alternation of small villages and areas of anthropic employment (quarries and forest harvest) and semi-naturalized forests. CAV is instead characterized by the full vegetation succession, from broad-leaf woodland and mixed forest to conifer and stands forest, shrubs, and alpine prairies. The area is relatively undisturbed, with few anthropic activities (traditional cattle husbandry and seasonal tourism). In CEV, small mammal monitoring was performed from 2019 to 2021 at monthly or bimonthly intervals during the winter (November–March) and summer (April, June, and August), respectively, in a mixed broad-leaved and coniferous forest at 1000 m a.s.l. In CAV, live trapping was performed in 2019 and 2020 at monthly intervals during the summer (May–October) along an altitudinal gradient (from 500 m a.s.l. to 2500 m a.s.l.), corresponding to the succession of alpine habitats (i.e., from broad-leaf woodland down the valley up to grasslands above the tree line).

Small mammals were captured using standard Ugglan Multiple Live Traps (model 2, Granhab, Sweden) set in a grid array, following a protocol that aimed at capturing especially rodents to compare assemblages across an altitudinal gradient. Capture–mark–recapture (CMR) techniques were adopted, and each animal was individually tagged with a subcutaneous Passive Integrated Transponder (PIT) tag (Trovan^®^ Ltd., Douglas, UK). At each capture event, we recorded information on individuals, such as species, PIT tag code, sex, body mass, breeding status, and ectoparasites load. Additionally, at first capture, an ear biopsy (using sterile disposable ear punch needles, Ø 3 mm; 20 mg) was collected for each animal and individually placed in tubes stored at −80 °C.

All animal handling procedures and ethical issues were approved by the Provincial Wildlife Management Committee (Prot. n. S044-5/2015/277268/2.4).

### 2.2. DNA Extraction, Amplification, and Sequencing

For DNA extraction, the ear samples (506 from CEV and 326 from CAV) were incubated overnight at 56 °C and isolated using the DNeasy 96 Blood & Tissue Kit (Qiagen, Hilden, Germany) according to the manufacturer’s instructions.

This study is part of a wider project investigating tick-borne pathogen circulation in small mammals. Therefore, the identification of *Hepatozoon* spp. was a bycatch while assessing the protocol for *Babesia* spp. detection on ear tissue samples. We applied a conventional polymerase chain reaction (PCR) method using the *Babesia* BJ1 5′–GTC TTG TAA TTG GAA TGA TGG–3′ and BN2 5′–TAG TTT ATG GTT AGG ACT ACG–3′ primers [46]. Indeed, beyond *Babesia* spp., BJ1 and BN2 primers can amplify the 18S RNA gene of some other protozoans [47], such as the 600-bp fragment of *Hepatozoon* spp. [48,49,50]. To discriminate the two parasites and detect coinfections, we analyzed all the samples separately (protocol of *Babesia* spp. not described) by specifically adapting the PCR reactions and temperature cycling profiles for each protozoan [47].

In particular, for *Hepatozoon* spp. detection, the PCR reactions were composed of 3-mM MgCl_2_, 0.1-mg/mL BSA, 0.2-mM each dNTP, 0.3-μM of each primer, 5× Flexi buffer, and 1.25-U/μL Go Taq Hot Start Polymerase (Promega) in 50 μL of total volume, including 1 μL of extracted DNA. After the initial denaturation at 95 °C for 2 min, 40 cycles of 95 °C for 20 s, 46 °C for 15 s, and 72 °C for 50 s were performed before a final incubation at 72 °C for 5 min. Negative controls, i.e., samples that did not contain DNA but only reagents, were included in each molecular essay to cross-check the quality for DNA extraction and PCR amplification. The success of amplification was confirmed using the Qiagen QIAxcel^®^ capillary electrophoresis system. Positive PCR products were purified using ExoSAP-IT (USB, Cleveland, OH, USA) according to the manufacturer’s instructions and then sequenced using Sanger sequencing. The DNA sequences were analyzed and aligned using Sequencher software version 5.4.6 (Gene Codes Corp., Ann Arbor, MI, USA) and Clustal X software version 2.0 [51] and subsequently compared with the data stored in the GenBank database with the Basic Local Alignment Search Tool (Blast; online version).

To verify the robustness of the PCR method, we also analyzed and sequenced double ear samples on a subset of individuals (N = 20, 2 samples from 10 individuals).

### 2.3. Statistical Analyses

The prevalence of *Hepatozoon* spp. was calculated for each rodent species with a 95% confidence interval (CI) using the EpiR package [52] in the R program [53]. We applied the Two Proportion Z-test to analyze the differences of the prevalence rates among rodent species. The differences were considered statistically significant if the *p*-values were <0.05.

Moreover, we investigated the role of the host species and environmental context on the probability of rodent infection with *Hepatozoon* spp., controlling for the life history traits. Specifically, we applied two analytical designs: (i) we evaluated the effect of anthropogenic pressure on rodent infection by comparing the study areas (CEV: high pressure; CAV: low pressure), only for a homogeneous altitude; (ii) we assessed how the probability of infection would vary across species along the alpine altitudinal gradient in a particularly wild context (CAV: 5 belts of 500 m, from 500 m a.s.l. to 2500 m a.s.l.). To this end, we fitted Generalized Linear Mixed Models (GLMMs; [54]) with binomial distribution of errors to the probability of infection of all samples with anthropogenic pressure and altitudinal belts, respectively, for the two designs, as the main covariates together with the species. We controlled for the sex and breeding status of the captured rodents (juveniles, subadults, and adult classes for both males and females). Finally, to account for spatial autocorrelation between captures, we included a grid as the most parsimonious random effect (Appendix A). Model selection was performed on the basis of the AICc score [55] (see Appendix A for details). We fitted the models using R software 4.1.2 [53] and packages *tidyverse* [56], *ppsr* [57], *MuMIn* [58], and *glmmTMB* [59].

In both proportion tests and in GLMMs, we discarded those sporadic species that were captured only a few times (<20 capture events).

## 3. Results

We analyzed 830 individual ear samples from 11 species (genetically determined through cytochrome-b [60]): 562 yellow-necked mice (*Apodemus flavicollis*), 10 wood mice (*Apodemus sylvaticus*), 24 snow voles (*Chionomys nivalis*), 2 common voles (*Microtus arvalis*), 2 pine voles (*Microtus subterraneus*), 1 short-tailed field vole (*Microtus agrestis*), 199 bank voles (*Myodes glareolus*), 25 common shrews (*Sorex araneus*), 3 alpine shrews (*Sorex alpinus*), 1 pygmy shrew (*Sorex minutus*), and 1 bicolored-shrew (*Crocidura leucodon*) (see Table S1). Additionally, 1790 ticks (1745 larvae and 45 nymphs) and 3782 ticks (3718 larvae and 64 nymphs) were counted on captured rodents in CEV and CAV, respectively. All sampled ticks belonged to the genus *Ixodes*. We also recorded the occurrence of other ectoparasites, i.e., fleas, and mites.

All accidentally caught shrews tested negative for *Hepatozoon* spp. and were therefore not included in the following analysis (see Table 1). The results from the 10 double samples were identical and confirmed the robustness of the PCR method.

Among rodents, for all samples, five species tested positive for *Hepatozoon* spp.: *A. flavicollis* (28.1%), *A. sylvaticus* (30.0%), *C. nivalis* (33.3%), *M. arvalis* (50%), and *My. glareolus* (58.8%) (see Table 1 for details), with an overall prevalence of 35.9% (see Appendix B for sequences).

**Figure 2:**
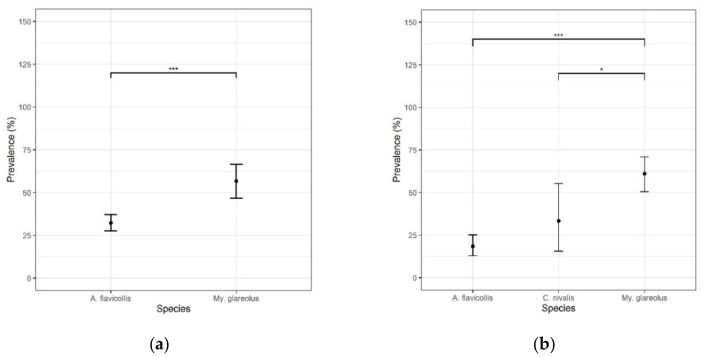
Difference of the prevalence rates of *Hepatozoon* spp. among rodent species from the Province of Trento, Italy (2019–2021): (**a**) in Cembra Valley (CEV) and (**b**) in Calamento Valley (CAV). Vertical bars represent the 95% confidence interval. Horizontal bars refer to significant differences, with *** = significance level < 0.001 and * = significance level < 0.05.

In Cembra Valley (CEV; 506 samples), *Hepatozoon* spp. was detected in all three rodent species captured (Table 1). *Hepatozoon* spp. prevalence was higher in *My. glareolus* if compared with *A. flavicollis* (Z-test, *p*-value = 6.855 × 10^−6^) (Figure 2a).

In Calamento Valley (CAV; 294 samples), four rodent species tested positive (Table 1). *My. glareolus* showed a statistically significant higher prevalence compared to *A. flavicollis* and *C. nivalis* (Z-test, *p*-value = 6.075 × 10^−12^ and *p*-value = 0.027, respectively) (Figure 2b). Among the species with too few captures to compute prevalence, *Hepatozoon* spp. was recorded in *M. arvalis*, in one individual out of the two captures and *A. sylvaticus*, in three out of eight captures.

The results of the GLMMs showed that rodent species have a crucial role in driving the infection of *Hepatozoon* spp. Conversely, both human pressure and the altitudinal gradient were not selected in the best models, indicating their scarce relevance in influencing the probability of becoming infected by *Hepatozoon* spp. (Appendix A). In particular, in the first analysis between study areas at 1000 m a.s.l., the infection probability depended on the species, with *My. glareolus* more likely to be infected than *A. flavicollis* (β = 1.07 ± 0.21; *p*-value = 3.27 × 10^−7^; reference category: *A. flavicollis*). In the second analysis, the infection of *Hepatozoon* spp. depended on the additive effect of the species and breeding status, confirming that *My. glareolus* was more prone to becoming infected if compared to *A. flavicollis* (β = 1.97 ± 0.30; *p*-value = 5.98 × 10^−11^; reference category: *A. flavicollis*), while no significant difference emerged between *A. flavicollis* and *C. nivalis* (Appendix A). In addition, a minor effect of the breeding status emerged, in which juveniles seemed to be marginally more infected compared to adults (β = 1.29 ± 0.70; *p*-value = 0.06; reference category: Adults), while we did not detect any significant difference between adults and subadults (Appendix A).

It is relevant to observe that, after sequencing, two samples (one from *S. araneus* and one from *My. glareolus*) previously assigned to *Hepatozoon* spp. were instead confirmed as *Babesia microti* (see Appendix B).

## 4. Discussion

This study reports for the first time the occurrence and prevalence of *Hepatozoon* spp. in wild rodents in the Italian Alps. In particular, we found a high prevalence in the most common woodland rodent hosts, i.e., *My. glareolus* and *A. flavicollis*, as well as in two other alpine species: the sympatric *A. sylvaticus* and *C. nivalis* at high altitudes.

This study is part of a wider project investigating tick-borne pathogen circulation in small mammals in the Italian Alps. In this context, the detection of rodent infection by *Hepatozoon* spp. represented a bycatch of the assessment of *Babesia* spp. For this reason, *Babesia*-specific primers were used for the screening, and the positive samples were further identified through sequencing. The identification of two positive samples of *Babesia microti* underlines the risk of mismatch when *Babesia*-specific primers are used for detecting *Hepatozoon* spp., as already underlined by other studies [47]. This finding suggests that follow-up sequencing is a compulsory step to discriminate this parasite by other protozoa when their detection is due to nonspecific protocols. For these reasons, we were not able to further identify *Hepatozoon* spp. at the species level due to the low quality of the obtained sequences. The identification could be refined by using *Hepatozoon*-specific primers (HEPF/HEPR [47]).

European studies on *Hepatozoon* spp. in wild rodents have been generally based on blood [32,47,61,62], organs [14,22,23], and, more rarely, on skin [13,63] samples, which are often collected through invasive sampling methods that, in many cases, require the suppression of the animals. In live rodents, *Hepatozoon* spp. can be identified only through blood sampling, to the best of our knowledge [31,32]. This comes with the drawback that traditional microscopy based on a blood smear can underestimate *Hepatozoon* spp. prevalence, especially with low intensity of infection [47]. On the contrary, PCR-based assays, such as those that we implemented using ear biopsy samples collected from live-trapped rodents, are considered more sensitive and robust. Coupling molecular screening approaches with less invasive sampling methods may accomplish conservation issues in cases of endangered and vulnerable species, e.g., *C. nivalis* [64], limiting the impact on the studied species. Within this framework, we believe that our contribution may provide a useful methodological approach. Although the comparison of past studies based on different protocols or matrices might not be feasible, the comparison between different procedures to assess *Hepatozoon* spp., e.g., by comparing ear tissue samples with skin ones as those collected in studies [13,63], could be an interesting direction of research.

The number of species of the small mammal community in which *Hepatozoon* spp. was detected, its first identification in *C. nivalis* (33.3%), and its occurrence at 2000 and 2500 m a.s.l., the highest elevation at which these parasites have ever been recorded, represent key findings to speculate on the transmission pathways of *Hepatozoon* spp. in the Alpine range. Additionally, this is the first record of *Hepatozoon* spp. in *A. sylvaticus* (30.0%) using molecular approaches [34,65]. Since small mammals can become infected from *Hepatozoon* spp. mainly though the ingestion of infected vectors, it is reasonable to consider *Hepatozoon* spp. as a vector-borne infectious agent for small mammal hosts and specifically in our case, although we did not assess the infective status and transmission dynamics of arthropod vectors. Our results suggest that *Hepatozoon* spp. are common in alpine environments, showing a high prevalence in a broad spectrum of alpine small mammal species. In particular, we did not detect any relevant difference in *Hepatozoon* spp. prevalence between anthropic and wild systems, implying a stable and ubiquitous presence of *Hepatozoon* spp. in the analyzed small mammal species. This is interesting, as *Hepatozoon* spp. have been previously studied mainly in domestic species [3,4,5,6,7,8]. The low host specificity displayed by *Hepatozoon* spp. [23,29] may indicate that these parasites persist in the environment sustained by competent and reservoir hosts, such as small mammals [66], both in wild and more anthropic settings. These conditions may increase the risk of spillover events among wildlife and domestic animals [67,68]. For example, *A. sylvaticus* generally shares food resources, part of the habitats and vectors, with other rodent competitor species, especially in case of high rodent and vector density [69], as it may occur in permissive environmental conditions (low elevations, in our case). The high density of reservoir hosts and of potentially infected vectors may amplify parasite circulation and spreading within the ecosystem, facilitating also the infection of less abundant host species [70]. This mechanism may have also favored the transition of the parasite to high altitudes, especially under increasingly milder climatic conditions. The less-limiting abiotic conditions along the altitudinal gradient occurring under climate change may promote an upward distributional shift of opportunistic small mammal host species (e.g., *My. glareolus* and *Apodemus* spp.) and, in turn, the survival and development of arthropod vectors (e.g., mites, fleas, and ticks) [27,71,72]. The combination of abiotic (i.e., climate) and biotic (i.e., interspecific interactions) conditions that permit host and vector persistence, together with the ability of a species to colonize favorable habitats, may therefore alter the local alpine communities, leading to novel host–vector species pairings and, thus, to the emergence of vector-borne diseases in new environments [73,74,75,76]. The occurrence of *Hepatozoon* spp. in *C. nivalis* and high altitudes that we recorded is compatible with these dynamics, although there is no track record of previous investigations at the same altitudes in small mammals (see Reference [28]).

The high rate of infection that we observed in different rodent species across a diversity of Alpine habitats may induce relevant cascading effects across the food web. In particular, being carnivore-competent hosts for *Hepatozoon* spp., a broad spectrum of both domestic (e.g., shepherd dogs [77]) and wild animals (e.g., snakes [78], meso- and large carnivores [20,79], and birds of prey [80]) may become infected through the ingestion of parasitized rodent preys. Specifically, small mammals are paratenic hosts for *Hepatozoon* spp., meaning that, despite not being necessary for parasite development, they help in maintaining its life cycle in the environment by supporting the cystozoite stages [40] infective for predators via ingestion [81]. The parasite can affect the immune response of predator secondary hosts, leading to population declines [39]. However, at the same time, since vector competence in becoming infected, replicating and transmitting the parasite varies across hosts [81], predators may represent dead end hosts for *Hepatozoon* spp. so indirectly reducing parasites transmission across ecosystem (i.e., dilution effect). This aspect of the transmission pathway of *Hepatozoon* spp. needs further investigation.

The prevalence of *Hepatozoon* spp. that we recorded in the most common rodent hosts, i.e., *My. glareolus* and *A. flavicollis*, is in partial accordance with other studies in Europe. In particular, we found that *My. glareolus* showed the highest prevalence of *Hepatozoon* spp. among all captured rodent species (58.8%), as was also observed in other studies [13,32,47,63], although with very variable levels (from 3.7% to 87.5% [13,23,47,48,62,82]). Conversely, the prevalence we found for *A. flavicollis* (28.1%) is higher than all previously published estimates that were below 10% [13,47,48,63] and may support a high prevalence of *Hepatozoon* spp. infection at lower elevations. The small sample size of *Microtus* spp. voles does not allow conclusive indications on the rate of infection of this genus (one positive *M. arvalis* out of five in total), as was the case also for other studies [23,31,32,47].

In conclusion, Apicomplexa remains one of the most poorly investigated groups among protozoa, so that accurate identification (via PCR-based methods coupled with sequencing) of *Hepatozoon* spp. in new wild hosts and habitats importantly contributes to the understanding of the ecological role of these parasites, especially under the current global change. Collectively, by detecting *Hepatozoon* spp. in a broad range of rodent hosts throughout the altitudinal gradient of Alpine habitats, we indicated the widespread occurrence of this parasite as likely supported by an expanding availability of arthropod vectors, due to variations in the temperature and moisture linked to climate change [75]. Therefore, this study represents a crucial starting point for future research combining ecological, epidemiological, and molecular analysis to evaluate the trophic transmission route of *Hepatozoon* spp. across the food chain in habitats particularly exposed to climate change, such as the Alpine range. In particular, studies investigating vector competence and distribution, as well as host ecology and their role in maintaining and transmitting *Hepatozoon* spp., are needed to clarify the function of this group of parasites in such changing ecosystems.

## Figures and Tables

**Table 1:** Prevalence of *Hepatozoon spp*. in Italy (2019–2021) from rodent and shrew specimens in two study sites (CEV = Cembra Valley; CAV = Calamento Valley) and across altitudes. CI = 95% Confidence Interval.

Family	Genus	Species	Study Site	PCR Positive/Total	% Prevalence (CI)	Altitude of Positives (m a.s.l.)
*Muridae*	*Apodemus*	*flavicollis*	CEV	127/394	32.23 (27.64–37.1)	1000
		*flavicollis*	CAV	31/168	18.45 (12.89–25.16)	500, 1000, 1500
		*sylvaticus*	CEV	3/8	37.5 (8.52–75.51)	1000
		*sylvaticus*	CAV	0/2	-	-
*Arvicolidae*	*Chionomys*	*nivalis*	CAV	8/24	33.33 (15.63–55.32)	2000, 2500
	*Microtus*	*agrestis*	CAV	0/1	-	-
		*arvalis*	CAV	1/2	50.00 (1.26–98.74)	2000
		*subterraneus*	CAV	0/2	-	-
	*Myodes*	*glareolus*	CEV	59/104	56.73 (46.65–66.41)	1000
		*glareolus*	CAV	58/95	61.05 (50.50–70.89)	1000, 1500, 2000
*Soricidae*	*Crocidura*	*leucodon*	CAV	0/1	-	-
	*Sorex*	*alpinus*	CAV	0/3	-	-
		*araneus*	CAV	0/25	-	-
		*minutus*	CAV	0/1	-	-
Total positive				287/830	34.58 (31.34–37.92)	
Total positive rodents				287/800	35.87 (32.55–39.30)	

**Figure A1:**
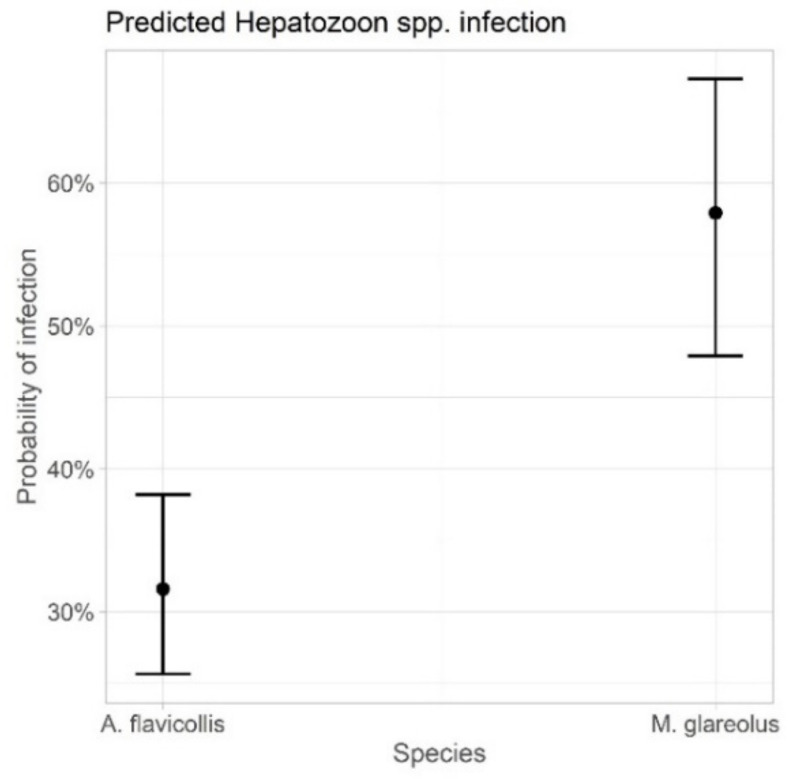
Predictive plot showing the effect of rodent species on probability of infection of *Hepatozoon* spp., after controlling for the effects of other covariates in the Generalized Linear Mixed Models (GLMMs; best model Table A2).

**Figure A2:**
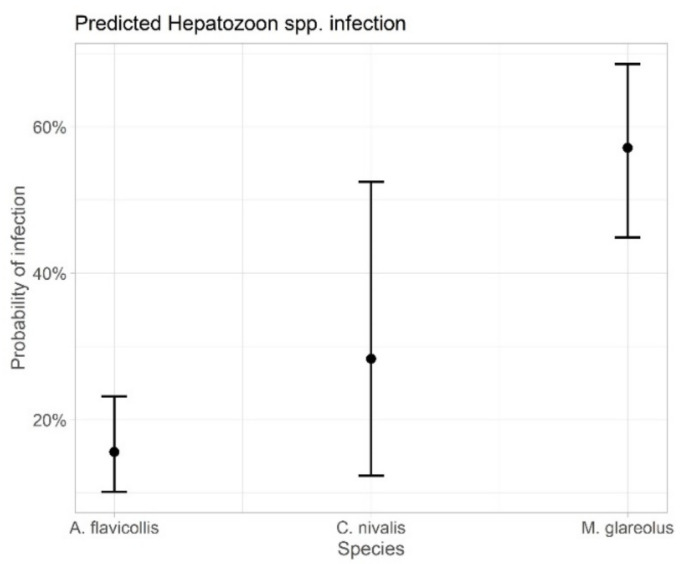
Predictive plot showing the effect of rodent species on probability of infection by *Hepatozoon* spp., after controlling for the effects of other covariates in the Generalized Linear Mixed Models (GLMMs; best model Table A5).

**Figure A3:**
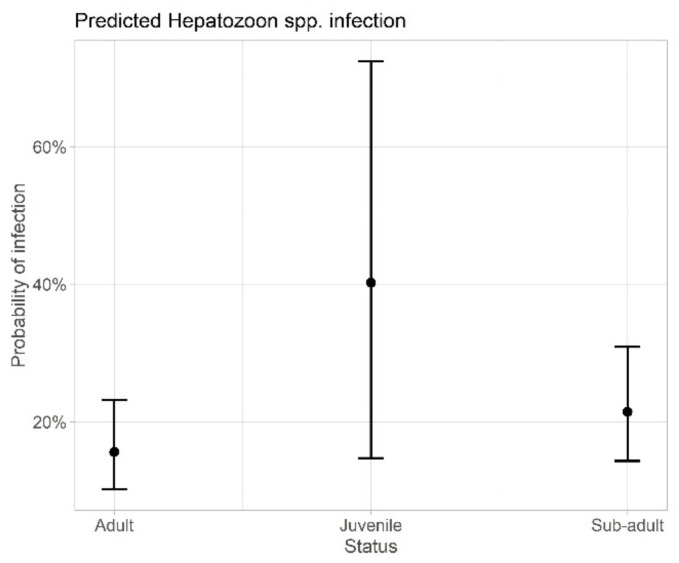
Predictive plot showing the effect of rodent status on probability of infection by *Hepatozoon* spp., after controlling for the effects of other covariates in the Generalized Linear Mixed Models (GLMMs; best model Table A5).

## Data Availability

The data presented in this study are available in the Supplementary Materials section and from the EuroSmallMammals database (https://eurosmallmammals.fmach.it (accessed on 13 February 2022)).

## Supplementary Materials

The following supporting information can be downloaded at https://www.mdpi.com/article/10.3390/microorganisms10040712/s1: Table S1: List of animals captured per site at Cembra and Calamento Valleys (Trento, Italy) and PCR results.

## Appendix A

To investigate the role of host species and environmental context (explanatory variables) on the probability of rodent infection with *Hepatozoon* spp. (response variable), a set of potentially biological meaningful covariates was considered, also controlling for life history traits, and specifically:

(i) life-history covariates
  1. Sex (**categorical variable**): Female or Male;
  2. Breeding status (**categorical variable**): Juvenile, Sub-adult, Adult;
  3. Species (**categorical variable**): Small mammal species captured ad least 20 times (i.e., *Apodemus flavicollis*, *Myodes glareolus*, *Chionomys nivalis* and *Sorex araneus*).
(ii) environmental covariates
  1. Study area (**categorical variable**): based on the anthropic pressure, Cembra Valley (CEV) is characterized as ‘high anthropic’ and Calamento Valley (CAV) as ‘low anthropic’.
  2. Altitude (**categorical variable**, only for CAV): the altitudinal gradient defined by 5 classes: 500 m, 1000 m, 1500 m, 2000 m and 2500 m a.s.l.

Specifically, we applied two analytical designs: (i), we evaluated the effect of anthropogenic pressure on rodent infection by comparing the study areas (Cembra: high pressure; Calamento: low pressure), only for altitudes homogeneous across the sites; (ii), we assessed how the probability of infection would vary across species along the alpine altitudinal gradient in a particularly wild context (Calamento Valley: 5 belts of 500 m from 500 m a.s.l. to 2500 m a.s.l.). To this end, we fitted Generalized Linear Mixed Models (GLMMs; [54]) with binomial distribution of errors to the probability of infection. To account for spatial autocorrelation of captures, we performed a first model selection on random effects based on second-order Akaike Information Criterion (AICc), by maximizing the fixed effects (i.e., considering the full model). In particular, we fitted model with random effect on trap nested within grid (‘Plot/Trap’), only on trapping grid (‘Plot’) and only on trap (‘Trap’) (Table A1, Table A4). After the selection of the most appropriate random effect [83], we performed a model selection on fixed effect starting from the full model, by ranking all possible model combinations derived from the full model on the basis of the AICc (Table A2, Table A5). The explanatory variables included in the full model were: small mammal species (‘Species’), breeding status (‘Status’), sex (‘Sex’), anthropogenic pressure characterizing the area (‘Area’; used only for first analysis) and altitudinal belts (‘Altitude’; used only for second analysis). The model with the lower AICc was selected as the best model [55] (Table A3, Table A6).

### Appendix A.1. Models across Study Areas with Different Anthropic Pressure

The most parsimonious model included ‘Species’ as fixed effect, and trapping grid as random effects (Table A1 and Table A2). The model coefficients revealed a strong positive effect of species and specifically, *Myodes glareolus* was more prone to becoming infected if compared with *Apodemus flavicollis* (Table A3; Figure A1). Conversely, the area and therefore the degree of anthropic pressure was not retained in the best model.

**Table A1:** Model selection according to AICc to identify the random effect. In bold the model selected. Legend: ‘Area’: anthropic pressure depending on study area; ‘Sex’: sex; ‘Status’: breeding status; ‘Species’: rodent species, ‘Plot’: trapping grid; ‘Trap’: trap station; Fixed effect’ = fixed effect modelled; ‘Random effect’ = random effects modelled; ‘AICc’ = AIC with a correction for small sample sizes; ‘∆AICc’ = relative differences between the fitted model and the Akaike ‘best-ranked’ model with the smallest AICc value; ‘weight’ = relative likelihood of a model.

Fixed Effect	Random Effect	AICc	∆AICc	Weight
**Area + Sex + Status + Species**	**1|Plot**	**766.2**	**0.00**	**0.71**
Area + Sex + Status + Species	1|Plot/Trap	768.3	2.06	0.25
Area + Sex + Status + Species	1|Trap	772.2	5.98	0.03

**Table A2:** Model selection to predict probability of infection of *Hepatozoon* spp., reporting the best model (in bold), the models retained within a ∆AICc ≤ 4, and the first model with ∆AICc > 4 (in italic). Legend: ‘Area’: anthropic pressure depending on study area; ‘Sex’: sex; ‘Status’: breeding status; ‘Species’: rodent species, ‘Plot’: trapping grid; ‘Trap’: trap station; Fixed effect’ = fixed effect modelled; ‘Random effect’ = random effects modelled; ‘AICc’ = AIC with a correction for small sample sizes; ‘∆AICc’ = relative differences between the fitted model and the Akaike ‘best-ranked’ model with the smallest AICc value; ‘weight’ = relative likelihood of a model.

Fixed Effect	Random Effect	AICc	∆AICc	Weight
**Species**	**1|Plot**	**752.72**	**0.00**	**0.41**
Area + Species	1|Plot	754.68	1.96	0.15
Sex + Species	1|Plot	754.74	2.03	0.15
Status + Species	1|Plot	755.12	2.41	0.12
Area + Species + Sex	1|Plot	756.71	4.00	0.05
*Area + Species + Status*	*1|Plot*	*757.05*	*4.33*	*0.05*

**Table A3:** Coefficients of the best model (Model 1, Table A2) fitting the probability of infection with *Hepatozoon* spp. The reference category for the covariate ‘Species’ is *A. flavicollis*. Legend: ‘Species’: rodent species; ‘Plot’: trapping grid; ‘τ00’ = method heterogeneity.

	Probability of Infection			
*Predictors*	*Estimate*	*Std. Error*	*Z Value*	*Pr*(>|*z*|)
Intercept	−0.77 ***	0.16	−4.86	1.15 × 10^−6^
Species *My. glareolus*	1.07 ***	0.21	5.11	3.27 × 10^−7^
Random effects				
τ00 Plot	0.12			
N trap	10			
Observations	590			
Marginal R^2^/Conditional R^2^	0.06/0.09			

*** *p* < 0.01.

### Appendix A.2. Models across Altitudinal Gradient in Calamento Valley

The most parsimonious model included ‘Species’ and ‘Status’ as fixed effects, and trapping grid as random effects (Table A4, Table A5). The model coefficients revealed a strong positive effect of species and a slightly positive effect of breeding status. Specifically, we confirmed that *Myodes glareolus* positively drove the infection of *Hepatozoon* spp. rather than *Apodemus flavicollis* and *Chionomys nivalis* (Table A6; Figure A2). Moreover, juvenile animals tended to be more infected compared to adults and sub-adults (Table A6; Figure A3). Conversely, elevation was not selected in the best model.

**Table A4:** Model selection according to AICc to identify the random effect. In bold the model selected. Legend: ‘Altitude’: elevation a.s.l.; ‘Sex’: sex; ‘Status’: breeding status; ‘Species’: rodent species, ‘Plot’: trapping grid; ‘Trap’: trap station; Fixed effect’ = fixed effect modelled; ‘Random effect’ = random effects modelled; ‘AICc’ = AIC with a correction for small sample sizes; ‘∆AICc’ = relative differences between the fitted model and the Akaike ‘best-ranked’ model with the smallest AICc value; ‘weight’ = relative likelihood of a model.

Fixed Effect	Random Effect	AICc	∆AICc	Weight
**Altitude + Sex + Status + Species**	**1|Plot**	**314.4**	**0.00**	**0.43**
Altitude + Sex + Status + Species	1|Plot/Trap	314.4	0.00	0.43
Altitude + Sex + Status + Species	1|Trap	316.6	2.19	0.14

**Table A5:** Model selection to predict probability of infection of *Hepatozoon* spp., reporting the best model (in bold), the models retained within a ∆AICc ≤ 4, and the first model with ∆AICc > 4 (in italic). Legend: ‘Altitude’: elevation a.s.l.; ‘Sex’: sex; ‘Status’: breeding status; ‘Species’: rodent species, ‘Plot’: trapping grid; ‘Trap’: trap station; Fixed effect’ = fixed effect modelled; ‘Random effect’ = random effects modelled; ‘AICc’ = AIC with a correction for small sample sizes; ‘∆AICc’ = relative differences between the fitted model and the Akaike ‘best-ranked’ model with the smallest AICc value; ‘weight’ = relative likelihood of a model.

Fixed Effect	Random Effect	AICc	∆AICc	Weight
**Species + Status**	**1|Plot**	**306.96**	**0.00**	**0.38**
Species	1|Plot	307.35	0.39	0.31
Sex + Species + Status	1|Plot	309.05	2.09	0.13
Sex + Species	1|Plot	309.41	2.45	0.11
*Altitude + Species*	*1|Plot*	*312.30*	*5.34*	*0.03*

**Table A6:** Coefficients of the best model (Model 1, Table A5) fitting the probability of infection with *Hepatozoon* spp. The reference category for the covariate ‘Species’ is *A. flavicollis*; the reference category for the covariate ‘Status’ is ‘Adult’. Legend: ‘Species’: rodent species; ‘Status’: breeding status; ‘Plot’: trapping grid; ‘τ00’ = method heterogeneity.

	Probability of Infection			
*Predictors*	*Estimate*	*Std. Error*	*Z Value*	*Pr*(>|*z*|)
Intercept	−1.69 ***	0.25	−6.75	1.47 × 10^−11^
Species *C. nivalis*	0.76	0.54	1.39	0.16
Species *My. glareolus*	1.97 ***	0.30	6.54	5.98 × 10^−11^
Status Juvenile	1.29 *	0.70	1.84	0.06
Status Sub-adult	0.39	0.30	1.31	0.19
Random effects				
τ00 Plot	1.15 × 10^−9^			
N trap	9			
Observations	272			
Marginal R^2^/Conditional R^2^	0.22/0.22			

* *p* < 0.1, *** *p* < 0.01.

## Appendix B

Among the 287 positive samples for *Hepatozoon* spp., we identified 151 entire high-quality sequences. After checking the mutations, four main different sequences emerged (TN-1–TN-4), which are repeated in all positive samples. The four different sequences were listed below. We also added the two sequences of *Babesia microti* (F-TN-5, F-TN-6) detected through *Hepatozoon* spp. protocol.

**>TN-1-Hep**

ACGGTATCTGATCGTCTTCGATCCCCTAACTTTCGTTCTTGATTAATGAA

AACATCTTTGGCAAATGCTTTCGCAGTAGTGTGTCTTTAACAAATCTAAG

AATTTCACCTCTGACAGTTAAATACAAATGCCCCCAACTGCTCCTATCAA

TCATTAATTTAGTTCTTAAAACCAATAACGTAGAACTAAAATCCTATTTT

ATTATTCCATGCTGCAGTATTCAAAACGTTAGCCTGCTTGAAACACTCTA

ATTTTCTCAAAGTAAAAATCCTCAAAATTGCATTCTACAATAAAGTAAAA

AACATTCCAAAGGACATTATTGCTAAAAACACACCAAGATACCACTCTTA

TAAATAAAAGCAGACCGGTTATTTCTAGCAAAAATTCAACTACGAGCTTT

TTAACTGCAACAATTTTAATATACGCTATTGGAGCTGGAATTACCGCGGC

TGCTGGCACCAGACTTGCCCTCCAATTGATACTTTAAAAAGTATTTAAAT

TT

**>TN-2-Hep**

ACGGTATCTGATCGTCTTCGATCCCCTAACTTTCGTTCTTGATTAATGAA

AACATCTTTGGCAAATGCTTTCGCAGTAGTGTGTCTTTAACAAATCTAAG

AATTTCACCTCTGACAGTTAAATACAAATGCCCCCAACTGCTCCTATCAA

TCATTAATTTAGTTCTTAAAACCAATAACGTAGAACTAAAATCCTATTTT

ATTATTCCATGCTGCAGTATTCAAAACGTTAGCCTGCTTGAAACACTCTA

ATTTTCTCAAAGTAAAAATCCTCAAAATTGCTTTTTACAATAAAGTAAAA

AACATTCCAAAGGATATTATTGCTAAAAGCACACCAAGATACCACTCTTA

TAAATAAAAGCAGACCGGTTATTTCTAGCAAAAATTCAACTACGAGCTTT

TTAACTGCAACAATTTTAATATACGCTATTGGAGCTGGAATTACCGCGGC

TGCTGGCACCAGACTTGCCCTCCAATTGATACTTTAAAAAGTATTTAAAT

TT

**>TN-3-Hep**

ACGGTATCTGATCGTCTTCGATCCCCTAACTTTCGTTCTTGATTAATGAA

AACATCTTTGGCAAATGCTTTCGCAGTAGTGTGTCTTTAACAAATCTAAG

AATTTCACCTCTGACAGTTAAATACAAATGCCCCCAACTGCTCCTATCAA

TCATTAATTTAGTTCTTAAAACCAATAACGTAGAACTAAAATCCTATTTT

ATTATTCCATGCTGCAGTATTCAAAACGTTAGCCTGCTTGAAACACTCTA

ATTTTCTCAAAGTAAAAATCCTGAAAATTGCTTCTCACAATAAAGTAAAA

AACATTTCAAAGGACATTATTGCTAAAAACACACCAAGATGCCACTTTTA

ATAATAAAAGCAGACCGGTTATTTTTAGCAAAAATTCAACTACGAGCTTT

TTAACTGCAACAATTTTAATATACGCTATTGGAGCTGGAATTACCGCGGC

TGCTGGCACCAGACTTGCCCTCCAATTGATACTTTAAAAAGTATTTAAAT

TT

**>TN-4-Hep**

ACGGTATCTGATCGTCTTCGATCCCCTAACTTTCGTTCTTGATTAATGAA

AACATCTTTGGCAAATGCTTTCGCAGTAGTGTGTCTTTAACAAATCTAAG

AATTTCACCTCTGACAGTTAAATACAAATGCCCCCAACTGCTCCTATCAA

TCATTAATTTAGTTCTTAAAACCAATAACGTAGAACTAAAATCCTATTTT

ATTATTCCATGCTGCAGTATTCAAAACGTTAGCCTGCTTGAAACACTCTA

ATTTTCTCAAAGTAAAAATCCTGAAAATTGCTTTTTACAATAAAGTAAAA

AACATTTCAAAGGACATTATTGCTAAAAACACACCAAGATACCACTTTTA

TTAATAAAAGCAGACCGGTTATTTTTAGCAAAAATTCAACTACGAGCTTT

TTAACTGCAACAATTTTAATATACGCTATTGGAGCTGGAATTACCGCGGC

TGCTGGCACCAGACTTGCCCTCCAATTGATACTTTAAAAAGTATTTAAAT

TT

**>F-TN-5_Babesia**

ACTACGACGGTATCTGATCGTCTTCGATCCCCTAACTTTCGTTCTTGATT

AATGAAAACATCCTTGGCAAATGCTTTCGCAGTAGTTCGTCTTTAACAAA

TCTAAGAATTTCACCTCTGACAGTTAAATACGAATGCCCCCAACTGCTCC

TATTAACCATTACTCTGGCTCAATAACCAACAAAATAGAACCAAAGTCCT

ACTTCATTATTCCATGCTGTAGTATTCAAGGCAAATGGCCTGTTTGAAAC

ACTCTAGTTTTCTCAAAGTAAATACTGGAAGATAGTAACCAATTAAGGAT

ACCAAATCCAGAAGATGCCAAGTCAATAAAATAACGCTCGAAAGCGAGAT

TAATGACAAGGCAGAAATTCAACTACGAGCTTCTTAACTGCAACAACTTT

AATATACGCTATTGGAGCTGGAATTACCGCGGCTGCTGGCACCAGACTTG

CCCTCCAATTGATACTCTGGGAAGGGTTTAGATTC

**>F-TN-6_Babesia**

ACTACGACGGTATCTGATCGTCTTCGATCCCCTAACTTTCGTTCTTGATT

AATGAAAACATCCTTGGCAAATGCTTTCGCAGTAGTTCGTCTTTAACAAA

TCTAAGAATTTCACCTCTGACAGTTAAATACGAATGCCCCCAACTGCTCC

TATTAACCATTACTCTGGCTCAATAACCAACAAAATAGAACCAAAGTCCT

ACTTCATTATTCCATGCTGTAGTATTCAAGGCAAATGGCCTGTTTGAAAC

ACTCTAGTTTTCTCAAAGTAAATACTGGAAGATAGTAACCAATTAAGGAT

ACCAAATCCAGAAGATGCCAAGTCAATAAAATAACGCTCGAAAGCGAGAT

TAATGACAAGGCAGAAATTCAACTACGAGCTTCTTAACTGCAACAACTTT

AATATACGCTATTGGAGCTGGAATTACCGCGGCTGCTGGCACCAGACTTG

CCCTCCAATTGATACTCTGGGAAGGGTTTAGATTC
